# Growth potentials of CCR5-tropic/CXCR4-tropic HIV-1mt clones in macaque cells

**DOI:** 10.3389/fmicb.2013.00218

**Published:** 2013-07-29

**Authors:** Naoya Doi, Ayaka Okubo, Mizumo Yamane, Yosuke Sakai, Akio Adachi, Masako Nomaguchi

**Affiliations:** Department of Microbiology, Institute of Health Biosciences, The University of Tokushima Graduate SchoolTokushima, Japan

Human immunodeficiency virus type 1 (HIV-1) is strictly adapted to humans, and cause AIDS only in humans. Consequently, no experimental animals susceptible to HIV-1 and suitable for the AIDS model study are available to date (Nomaguchi et al., 2008, 2011). To overcome this issue, viruses genetically related to HIV-1 have been challenged into macaque monkeys to mimic the natural HIV-1 infection. Viruses used for these experiments are simian immunodeficiency viruses (SIVs), SIVs chimeric with parts of HIV-1 sequences (SHIVs), and macaque tropic HIV-1 derivatives carrying a small portion of SIV genome (HIV-1mt clones). Because viruses of the SHIV and HIV-1mt groups carry HIV-1 genes/sequences, their scientific/medical significance and impact are evident. Although some SHIVs indeed induce AIDS in macaques, accumulating evidences have demonstrated that the genuine CCR5-tropism of input viruses is prerequisite for superimposing the experimental outcome on the natural disease progression in humans (Feinberg and Moore, 2002; Margolis and Shattock, 2006). Therefore, a number of CCR5-tropic SHIVs currently have been generated and utilized for *in vivo* macaque experiments (Hsu et al., 2003; Humbert et al., 2008; Nishimura et al., 2010; Fujita et al., 2012).

**Figure 1:**
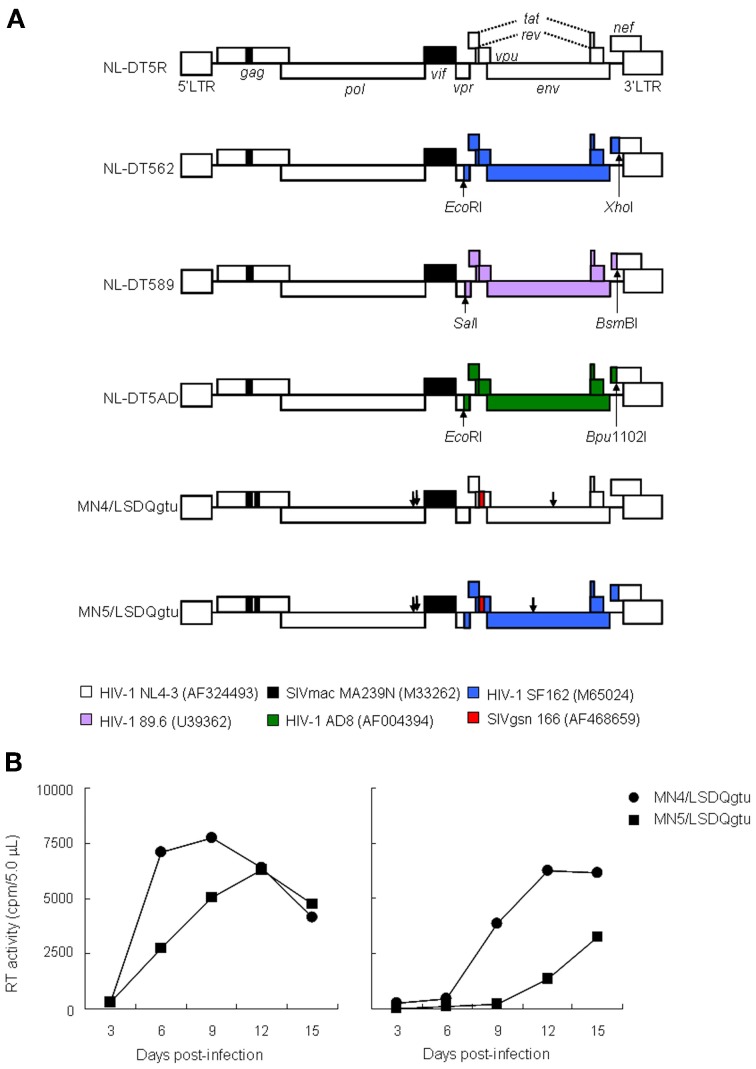
**Characteristics of HIV-1mt clones. (A)** Proviral genome structure of various HIV-1mt clones. Genomes of various HIV-1mt clones generated in our laboratory are schematically illustrated. White and black (a small portion of *gag*-capsid and an entire *vif*) areas stand for the genomic regions of HIV-1 NL4-3 (Adachi et al., 1986) and SIVmac MA239N (Shibata et al., 1991; Doi et al., 2010), respectively. The other colored regions are the sequences derived from various SIV/HIV-1s as shown. Restriction enzyme sites used for the insertion of these sequences into NL-DT5R are also shown. GenBank accession numbers are indicated in parentheses. Arrows represent the adaptive mutation sites in *pol*-integrase and *env*-gp120 that enhance virus growth potentials (Nomaguchi et al., 2013b). While NL-DT5R and MN4/LSDQgtu are CXCR4-tropic viruses, NL-DT562, NL-DT5AD, and MN5/LSDQgtu are CCR5-tropic. Although examined in macaque cells, the growth ability of NL-DT589 is not yet proven (see text). mac, rhesus macaques; gsn, greater spot-nosed monkeys. **(B)** Viral replication kinetics in M1.3S cells. Cell-free viruses were prepared from 293T cells transfected with proviral clones indicated, and equal amounts [5 × 10^6^ and 5 × 10^5^ reverse transcriptase (RT) units for left and right panels, respectively] were inoculated into M1.3S cells (2 × 10^6^ cells). Viral replication was monitored at intervals by RT activity in the culture supernatants. The experiments were done as described previously (Kamada et al., 2006). M1.3S is the most refractory cell line to infection with SIVmac/HIV-1mt clones to the best of our knowledge, but is CD4-, CXCR4-, and CCR5-positive. NL-DT5R and NL-DT562 do not grow at all in M1.3S cells.

Recently, prototype HIV-1mt clones, CXCR4-tropic NL-DT5R, and dual-tropic (CXCR4- and CCR5-tropic) stHIV-1, have been generated by us (Kamada et al., 2006) and others (Hatziioannou et al., 2006), respectively. We selected three distinct Env sequences and made three proviral constructs in the backbone of the NL-DT5R genome to obtain CCR5-tropic/dual-tropic viruses (Figure 1A), based on the published results (Hsu et al., 2003; Hatziioannou et al., 2006; Matsuda et al., 2010; Nishimura et al., 2010). Of the three clones constructed, while NL-DT562 grew in a cynomolgus macaeque cell line HSC-F (Akari et al., 1996; Fujita et al., 2003), the other two viruses designated NL-DT589 and NL-DT5AD did not (Doi et al., 2010; our unpublished observations). The replication efficiency in HSC-F cells of NL-DT562 was much lower than that of the parental virus NL-DT5R (Doi et al., 2010). When examined in CD8^+^ cell-depleted pig-tailed macaque peripheral blood mononuclear cells (PBMCs), NL-DT5AD was found to be replication-competent in addition to NL-DT562 (Igarashi and Adachi, unpublished results). However, NL-DT5AD grew more poorly than NL-DT562, and NL-DT562 itself propagated much more inefficiently again than NL-DT5R in these PBMCs. Of note, NL-DT562 was confirmed to use CCR5 for cell entry (our unpublished data). To improve the replication ability of NL-DT562, we extensively modified its genome by adaptation to macaque cells and also by *in vitro* mutagenesis (Nomaguchi et al., 2008, 2011, 2013a,b; Nomaguchi et al., submitted). As a result, the same mutations were introduced into the corresponding genomic regions of NL-DT5R and NL-DT562 encoding Gag-capsid, Pol-integrase, and Vpu-transmembrane domain. Numerous growth-enhancing adaptive mutations were found to separately occur in the Env of NL-DT562, but only one in the Env of NL-DT5R (Nomaguchi et al., 2013b). Since the enhancement of virus growth by these mutations is strictly Env sequence-dependent (Nomaguchi et al., 2013b), only a single best mutation for viral replication was introduced into the *env* gene of each clone. As shown in Figure 1B, the final version of CCR5-tropic virus currently constructed (MN5/LSDQgtu in Figure 1A) surely grew extremely better than NL-DT562 in a rhesus macaque cell line M1.3S (Doi et al., 2011), but more poorly relative to MN4/LSDQgtu (Nomaguchi et al., submitted) (Figure 1A), a CXCR4-tropic virus derived from NL-DT5R (a virus corresponding to MN5/LSDQgtu). Taken all together, we are unable yet to have a CCR5-tropic HIV-1mt clone that grows better or equally well in macaque cells relative to CXCR4-tropic MN4/LSDQgtu. Virological and molecular basis for this negative result is presently unknown, but it is certain that the Env sequence is important for viral growth potentials. Extensive search for appropriate Env sequences to confer CCR5-tropism and high replication-ability on HIV-1mt clones is required for our final purpose, i.e., the generation of proviral clones virologically similar to viruses of the SIVmac group that are pathogenic for macaques. In this regard, it is tempting to use “intracellular homologous recombination” as a measure to readily generate recombinant HIV-1 clones (Fujita et al., 2012).

Despite the every effort of researchers, so far, no appreciable disease was induced in pig-tailed and cynomolgus macaques infected with various HIV-1mt clones (Igarashi et al., 2007; Hatziioannou et al., 2009; Saito et al., 2011, 2013; Thippeshappa et al., 2011). Although the rhesus macaque is thought to be the best macaque species for infection experiments of this kind from various virological and primatological points of view, no attempts to infect it with HIV-1mt clones have been made to date, probably due to its highly resistant nature to the viruses. Common characteristics of the non-morbifical infections as described above are low viral loads relative to those in pathogenetic infections with SIV/SHIV/HIV-1 and no apparent viral set points in the course of infection. Without initial burst of viruses in hosts to guarantee viral amount and diversity enough for persistent infection, viruses may not survive in individuals/populations. Further improvement of the replication ability of CCR5-tropic HIV-1mt clones would be necessary to establish HIV-1mt/macaque model systems, the rhesus system in particular, for natural infections of HIV-1, and finally for human AIDS research.
